# Analysis of cytokines in SARS-CoV-2 or COVID-19 patients in Erbil city, Kurdistan Region of Iraq

**DOI:** 10.1371/journal.pone.0250330

**Published:** 2021-04-29

**Authors:** Mohammed Yousif Merza, Rundk Ahmed Hwaiz, Badraldin Kareem Hamad, Karzan Abdulmuhsin Mohammad, Harmand Ali Hama, Abdulkarim Yasin Karim

**Affiliations:** 1 Clinical Analysis Department, College of Pharmacy, Hawler Medical University, Erbil, Kurdistan Region, Iraq; 2 Medical Analysis Department, College of Science, Tishk International University, Erbil, Kurdistan Region, Iraq; 3 Department of Clinical Biochemistry, College of Health Science, Hawler Medical University, Erbil, Kurdistan Region, Iraq; 4 Department of Pharmacy, Knowledge University, Erbil, Kurdistan Region, Iraq; 5 Research Center, Salahaddin University-Erbil, Erbil, Kurdistan Region, Iraq; 6 Department of Biology, Faculty of Education, Tishk International University, Erbil, Kurdistan Region, Iraq; 7 Department of Biology, College of Science, Salahaddin University-Erbil, Erbil, Kurdistan Region, Iraq; Emory University School of Medicine, UNITED STATES

## Abstract

The emergence of the novel coronavirus and then pandemic outbreak was coined 2019- nCoV or COVID-19 (or SARS-CoV-2 disease 2019). This disease has a mortality rate of about 3·7 percent, and successful therapy is desperately needed to combat it. The exact cellular mechanisms of COVID-19 need to be illustrated in detail. This study aimed to evaluate serum cytokines in COVID-19 patients. In this study, serum was collected from volunteer individuals, moderate COVID-19 patients, severe cases of COVID-19 patients, and patients who recovered from COVID-19 (n = 122). The serum concentrations of interleukins such as IL-1, IL-4, IL-6, IL-8, IL-10, and tumor necrosis factor-alpha (TNF-α), were measured by enzyme-linked immunosorbent assays (ELISA). The concentrations of IL-1 and TNF-α were did not differ significantly among groups. However, the concentration of IL-6 was significantly higher in moderate COVID-19 and severe cases of COVID-19 groups compared to control and recovered groups indicating it to be an independent predictor in the coronavirus disease. The levels of IFN-γ and IL-4 were significantly lower in the recovery group than the severe case of the COVID-19 group. In contrast, the level of IL-10 in recovered COVID-19 patients was significantly higher in compare to severe cases, COVID-19 patients. Varying levels of cytokines were detected in COVID-19 group than control group suggesting distinct immunoregulatory mechanisms involved in COVID-19 pathogenesis. However, additional investigations are needed to be to be performed to understand the exact cellular mechanism of this disease.

## Introduction

The novel coronavirus disease (COVID-19), also initially named as SARS-CoV-2 Disease 2019, due to its similarity with SARS-CoV has a mortality rate of about 3·7 percent, and effective therapies are urgently needed to combat it [1]. The World Health Organization (WHO) has acknowledged and declared novel coronavirus as the infectious agent which leads to acute respiratory syndrome (ARS) [2]. However, no clear and appropriate treatment for COVID-19 is found since the pathogenesis of COVID-19 is incomprehensible. The major clinical feature of COVID-19 is brought about by causing acute injuries in the lungs, which leads to acute respiratory distress syndrome (ARDS) in most severe ARS patients. Inflammatory, immune cells, cytokines, and adhesion molecules are crucial factors involved in acute lung injury. Previous studies have shown some pro-inflammatory cytokines to play a crucial role throughout acute lung injury, for instance, acute pancreatitis and sepsis [3, 4]. They also demonstrated that infection with viral agents causes upregulation of cytokines such as Tumor Necrosis Factor alpha (TNF-α), which is considered as an important mediator of inflammation [5, 6]. However, the pathways for the novel ARDS-induced coronavirus infection remains yet to be elucidated. A recent study suggests that hyperinflammation in SARS-CoV-2 patients is due to cytokines storm, especially in immunosuppressed patients, and that, screening and managing these cytokines could improve the mortality rate [7]. Therapeutic strategies for SARS-CoV-2 could comprise of antibiotics, intravenous immunoglobulin (Ig), selective blockage of cytokines, and Janus Kinase (JAK) inhibition [8, 9].

Cytokines are well known inflammatory mediators that enhance the inflammatory responses, such as tumor growth factor-beta (TGF-β), gamma interferon (IFN- γ), IL-1, IL-8, IL-6, TNF-α, while some other cytokines function to reduce the inflammation like IL-4 and IL-10 [10]. In the early stage of SARS, the T-lymphocytes counts were low, especially in severe cases, indicating a change in immunomodulatory function [11]. Therefore, it becomes important to analyse the cytokine load in the sera of patients with COVID-19, in comparison to healthy and recovered individuals in order to decipher the cellular mechanism behind the pathogenesis of coronavirus.

In this study, the levels of TNF-α, IL-1α, IL-4, IL-6, IL-8, and IL-10, were measured through ELISA in sera from healthy volunteers as a control group, patients with moderate COVID-19, patients with severe COVID-19, and patients who had recovered from COVID-19.

## Materials and methods

Ethic Permission Number: HMU-PH-EC 170320 by Hawler Medical University ethic committee.

Three public hospitals in the Erbil city of Kurdistan, Iraq, namely Rizgari, Peshmarga, and Emirate were converted into COVID-19 treatment centers. A total of 128 patients, with confirmed COVID-19 between March to June 2020 were enrolled in this study. All patients were diagnosed and confirmed to be positive for COVID-19 by Real Time-Polymerase Chain Reaction (RT-PCR) in the central laboratory-Erbil.

The patients with COVID-19 were grouped into moderate cases of COVID-19, severe cases of COVID-19, and recovered cases from COVID-19 between male and females according to intensive care, complications and number of deaths (Table 1). Blood samples were collected to obtain serum from COVID-19 patients, three to seven days after symptoms developed. Following this, patients received medication such as Hydroxychloroquine (200 mg/day) and Favipiravir (200 mg/day). In severe COVID-19 case group whose serum samples were collected, ten of the patients were treated with hydroxychloroquine and Favipiravir for seven days before sampling. In the control group, blood samples were obtained from healthy individuals. The sampling was done under sterile conditions. The samples were centrifuged at 800g, 4°C for 10 min and the supernatant was stored at -80°C. Serum levels of IL-1α, IL-4, IL-6, IL-8, IL-10, TNF-α, and IFN-γ in the samples were measured by Enzyme Linked Immunosorbent Assay (ELISA) using Quantikine kits (Thermos Fisher Scientific) at wavelength of 450 nm. Manufacturer’s protocol was followed. The minimum concentration that could be detected by the kit was 10 pg/mL.

**Table 1:** Patient disposition in the study groups (n = 122).

	Number of patients			Ages		Intensive Care Unit	Complication	No. of deaths
Group	*(n)*	Male (M)	Female (F)	Range	Mean			
Control	25	18	7	23–54	23.65			
Moderate COVID-19	41	29	12	15–76	35.7	No	No	0
Severe cases of COVID-19	15	6	-	30–81	51.75	Yes	No	6
Recovered from COVID-19	41	22	19	17–73	27.3	No	No	0

The classification into mild, moderate and severe in the COVID-19 group depended on factors as mentioned here: Mild cases are usually with normal oxygen saturation and normal haematological and biochemical tests. Moderate cases are those with abnormal biochemical and or haematological test results with oxygen saturation between 80–90% and they were put on oxygen. Severe cases are those with abnormal biochemical and haematological test with very low oxygen saturation (<80) and they were either on oxygen, and or Continuous Positive Airway Pressure (CPAP).

### Statistical analysis

Univariate analysis was used to compare patients with normal and abnormal serum cytokine activities. A *P*-value of less than 0.05 was considered significant and n represents the total number of patients. Statistical analysis was performed by IBM SPSS Statistics software version 20 and data were reported as mean ± SD unless otherwise indicated.

## Results and discussion

In the current study, we evaluated the serum cytokine levels in COVID-19 patients in Erbil city of Iraq. The patient disposition in the enrolled patients has been presented in Table 1. There were 25, 41, 15, 41 in the control, moderately affected COVID-19 patients, severely affected COVID-19 patients and recovered COVID-19 patients respectively.

Serum concentrations of TNF- α and IL-1α in different groups of COVID-19 patients were evaluated. The TNF-α concentration in control, moderate COVID-19, severe cases of COVID-19, and recovered from COVID-19 groups were 86.3±5.2, 78.7±5.2, 68.3±6.2, and 57.9±3.8 pg/ml, respectively and did not vary significantly (P>0.05) (Table 2). The concentration of serum IL-1α in control, moderate COVID-19, severe cases of COVID-19, and recovered from COVID-19 groups were 6.24±2.32, 4.61±3.52, 4.45±7.31, and 4.31±2.31 pg/ml, respectively. The levels of IL-1α in moderate COVID-19, severe cases of COVID-19, and recovered from COVID -19 groups did not vary significantly different as compared with the control group (*P*>0.05). TNF-α and IL-1 have been demonstrated to play a key role in the pathogenesis of Acute Lung Injury (ALI) [12]. It has been reported that the serum concentration of TNF-α increased in some patients infected with the coronavirus in the respiratory system [13]. However, herein, the serum concentrations of TNF- α and IL-1α in moderate and severe cases of COVID-19 patients showed an insignificant increase, suggesting that the response of host immune system might be different from SARS-CoV-2 and could be due to infections from other pathogens.

**Table 2:** Concentration of cytokines in the sera of enrolled participants (n = 122).

Groups	*n*	TNF-α (pg/ml)	IL-1α (pg/ml)	IL-6 (pg/ml)	INF-γ (pg/ml)	IL-8 (pg/ml)	IL-4 (pg/ml)	IL-10 (pg/ml)
**Control**	25	86.3 ± 5.2	6.24 ± 2.32	54.8 ± 8.2	84.12 ± 45.4	274 ± 68	96 ± 94	71.2 ± 3
**Moderate COVID-19**	41	78.7 ± 5.2	4.61 ± 3.52	197.4 ± 93^#^	76.3 ± 22.1	198 ± 21^#^	108 ± 26	74.3 ± 6.2
**Severe cases of COVID-19**	15	68.3 ± 6.2	4.45 ± 7.31	497.3± 421^#^	88.23 ± 22.1	151 ± 21^#^	93 ± 21	61.1 ± 2.1
**Recovered from COVID-19**	41	57.9 ± 3.8	4.31 ± 2.31	82.4 ± 45.7	43.7 ± 11.2^#^	249 ± 130	70 ± 2^#^	159 ± 62^#^

The data are presented as mean ± standard deviation. *P* value < 0.05 for a comparison with the control group

# denotes significant difference

The serum concentration of IL-6 were elevated in COVID-19 patients. The IL-6 levels were 54.8±8.2, 197.4±93, 497.33±422, and 82.4±45.7 pg/ml in control, moderate, severe, and recovered patient group, respectively. This level was significantly higher in moderately and severely affected COVID-19 patients as compared with the control group. However, no significant differences were detected between controls and recovered from the COVID-19 group, suggesting that the severity of IL-6 was positively co-related to the severity of COVID-19. Previous studies have shown that IL-6 induces C-reactive protein (CRP), fibrinogen, serum amyloid A (SAA), and hepcidin in hepatocytes. IL-6 are produced by leukocytes in the blood or by exact in the site of injury tissue as endothelial cells, fibroblasts, or alveolar epithelial cells [14, 15]. Electron microscopy examination have shown coronavirus particles to be present in the cytoplasm of epithelial cells of infected patients suggesting epithelial cells of bronchial and alveolar regions to be the primary target cells of this virus [16]. We, therefore, hypothesize that the increased serum concentration of IL-6 in the moderate and severe cases of the COVID-19 group might be due to the affected epithelial cells of the bronchial and alveolar regions. This hypothesis could be further strengthened if higher IL-6 concentrations are detected in Broncho-alveolar lavage fluid of the COVID-19 patients. Moreover, the IFN-γ concentrations were 84.12±45.4, 76.3±22.1, 88.23±22.1, and 43.7±11.2 pg/ml in the control, moderate COVID-19, severe cases of COVID-19, and recovered from COVID-19 groups, respectively and did not vary significantly among groups.

Serum IL-8 levels were found to be low in COVID-19 patients. The IL-8 levels were 274±68, 198±21, 151±21, and 249±130 pg/ml in control, moderate, severe and recovered COVID-19 patient groups, respectively. Data shows that IL-8 levels in moderate and severe COVID-19 cases were significantly lower compared with the control group. However, no significant differences were found between the control group and recovered from the COVID-19 group, suggesting IL-8 responses peak during the COVID-19 infection when the viral load is high, and subsides as patients recover from the infection. A previous study has shown that the CD4 and CD8 subset of T-Lymphocytes decrease in patients with early-stage of SARS [17]. This decline might suggest the suppression of T-lymphocyte dependent immune responses. Subsequently, the significant decrease in IL-8 levels in COVID-19 patients may be due to the depletion of T lymphocytes depleted in these patients.

Serum concentration of IL-4 were 96 ± 94, 108 ± 26, 93 ± 21, and 90 ± 2 pg/ml in the control, moderate COVID-19, severe COVID-19, and recovered COVID-19 groups, respectively. The significant change of serum concentration of IL-4 was only detected in recovered from COVID-19 group but not in the patients with COVID-19 or severe cases of COVID-19 compared to the control group, suggesting the role of Th2 mediated responses in recovery. The serum concentrations of IL-10 were 71.2 ± 3, 74.3 ± 6.2, 61.1 ± 2.1, and 159 ± 62 pg/ml, respectively. Thus, the serum concentration of IL-10 was significantly (P<0.05) higher in recovered patient group compared to control, moderate and severe cases of COVID-19 groups. IL-10 has been shown to inhibit TNF-α and neutrophil activation in acute lung injury [18]. The increased serum IL-10 levels in recovered patients may reflect certain protective mechanisms exerted by IL-10; however, further studies are required to show the precise mechanism of these protection.

Our study is in close corroboration with the studies performed by Chen et al who demonstrated an increase in the IL-6 levels, but the concentration of TNF-α, IL-1, IL-8, and IL-10 remained unaltered in severely affected patients [19]. This is however in contrast with some other studies carried out by Diane et al and Zhang et al who showed increases in both IL-6 and IL-8. Diane et al suggested that IL-6 and TNF-α can be independent predictors of the disease severity [5, 12]. A recent review also highlighted the inevitable cytokine storm of IL-6 in severe COVID-19 [20]. The difference in the IL-8 and TNF-α could be due to the difference in timing of sampling, specific stage of coronavirus lifecycle in the host and differences in the disease severity. This is however subject to further research. The main pathological characteristic of SARS-CoV-2 was acute lung injury. This lung injury might be activated by coronavirus or other pathogens, which leads to release of cytokines by the downstream inflammatory and immune pathways [4, 5].

### Effect of treatment on serum cytokines

For evaluating the impact of hydroxychloroquine and Favipiravir treatment on the levels of the cytokine in serum from SARS-Cov-2 patients, the levels of cytokines in patients who received treatments were measured. Statistical analysis revealed no significant differences for any of the cytokine level in both moderate and severe groups (Table 3).

**Table 3:** Effect of hydroxychloroquine and Favipiravir treatments on serum cytokines.

Groups	*n*	TNF-α (pg/ml)	IL-1α (pg/ml)	IL-6 (pg/ml)	TNF-γ (pg/ml)	IL-8 (pg/ml)	IL-4 (pg/ml)	IL-10 (pg/ml)
Control	10	66.2 ± 8.6	11.24 ± 1.6	63.8 ± 8.4	73.3 ± 34.4	248 ± 38	84.4 ± 42	63.2 ± 4
Moderate-COVID-19+Treatment	10	59.1 ± 9.4	9.16 ± 6.3	143.4 ± 44	85.1 ± 27.3	206 ± 12	97.54 ± 15	64.6 ± 11
Moderate-COVID-19 without treatment	10	51.6 ± 11.2	8.11 ± 4.3	324.3± 421#	78.9 ± 12.6	174 ± 32#	81.3 ± 27	63.8 ± 6.5

The data are means ± standard errors. *P* value < 0.05 for a comparison with the control group, n = number of patients

Since some patients with COVID-19 were treated with hydroxychloroquine and favipiravir while obtaining blood samples, it was important to examine the possible impact of the hydroxychloroquine on serum cytokine levels. Moderate COVID-19 patients and severe COVID-19 patients were classified into two groups, one containing treated patients and one containing untreated patients. Our findings indicate no statistical differences in serum cytokines between moderate COVID-19 with or without treatment (Tables 3 and 4). This indicated that short treatment had no impact on circulating cytokine levels in these patients with COVID-19. Analysis of cytokines in COVID-19 patients’ bronchoalveolar lavage fluid is necessary because it might help us in the determination whether the cytokines are involved in the pathology in injured lung tissue.

**Table 4:** Effect of hydroxychloroquine and Favipiravir treatments on serum cytokines.

Groups	*n*	TNF-α (pg/ml)	IL-1α (pg/ml)	IL-6 (pg/ml)	TNF-γ (pg/ml)	IL-8 (pg/ml)	IL-4 (pg/ml)	IL-10 (pg/ml)
Control	10	45.2 ± 5.25	14.43 ± 2.3	84.2 ± 9.3	54.3 ± 9	18.7 ± 54	64 ± 46	85.5 ± 6.2
Severe case of COVID-19 +Treatment	10	54.3 ± 12.3	11.8 ± 5.6	211.6 ± 63#	75.8± 33	192.8 ± 26#	86.7 ± 22	73.4 ± 8
Severe cases of COVID-19 without treatment	10	48.4 ± 6.6	15.2 ± 6.9	287.6± 234#	66.4 ± 18	144.2 ± 32#	76.6 ± 18	91.6 ± 4

The data are means ± standard errors. *P* value < 0.05 for a comparison with the control group.

## Conclusion

The changes in serum cytokines with SARS-CoV-2 indicated the host’s immune responses against the coronavirus inflammation seem to be different from what has been seen with other viral pathogens. The serum concentration of IL-6, IL-8 and IL-10 might be considered as a reflective sign of the COVID-19 severity. Besides, these findings indicate different immunoregulatory events during and after SARS-CoV-2 infection, which may contribute to our understanding of the pathogenesis of this disease.
